# Molecular characterization of circulating colorectal tumor cells defines genetic signatures for individualized cancer care

**DOI:** 10.18632/oncotarget.19138

**Published:** 2017-07-10

**Authors:** Say Li Kong, Xingliang Liu, Nur-Afidah Mohamed Suhaimi, Kenneth Jia Hao Koh, Min Hu, Daniel Yoke San Lee, Igor Cima, Wai Min Phyo, Esther Xing Wei Lee, Joyce A. Tai, Yu Miin Foong, Jess Honganh Vo, Poh Koon Koh, Tong Zhang, Jackie Y. Ying, Bing Lim, Min-Han Tan, Axel M. Hillmer

**Affiliations:** ^1^ Genome Institute of Singapore, Singapore 138672, Singapore; ^2^ Institute of Bioengineering and Nanotechnology, Singapore 138669, Singapore; ^3^ German Cancer Consortium (DKTK), Essen/Düsseldorf, Heidelberg 69120, Germany; ^4^ German Cancer Research Center (DKFZ), Heidelberg 69120, Germany; ^5^ Lucence Diagnostics Pte Ltd, Singapore 159555, Singapore; ^6^ Concord Cancer Hospital Singapore, Singapore 289891, Singapore; ^7^ TRON-Translational Oncology at the University Medical Center of The Johannes Gutenberg University gGmbH, Mainz 55131, Germany

**Keywords:** circulating tumor cells, colorectal cancer, targeted sequencing, druggable mutation, mutation signatures

## Abstract

Studies on circulating tumor cells (CTCs) have largely focused on platform development and CTC enumeration rather than on the genomic characterization of CTCs. To address this, we performed targeted sequencing of CTCs of colorectal cancer patients and compared the mutations with the matched primary tumors. We collected preoperative blood and matched primary tumor samples from 48 colorectal cancer patients. CTCs were isolated using a label-free microfiltration device on a silicon microsieve. Upon whole genome amplification, we performed amplicon-based targeted sequencing on a panel of 39 druggable and frequently mutated genes on both CTCs and fresh-frozen tumor samples. We developed an analysis pipeline to minimize false-positive detection of somatic mutations in amplified DNA. In 60% of the CTC-enriched blood samples, we detected primary tumor matching mutations. We found a significant positive correlation between the allele frequencies of somatic mutations detected in CTCs and abnormal CEA serum level. Strikingly, we found driver mutations and amplifications in cancer and druggable genes such as *APC, KRAS, TP53, ERBB3*, *FBXW7* and *ERBB2*. In addition, we found that CTCs carried mutation signatures that resembled the signatures of their primary tumors. Cumulatively, our study defined genetic signatures and somatic mutation frequency of colorectal CTCs. The identification of druggable mutations in CTCs of preoperative colorectal cancer patients could lead to more timely and focused therapeutic interventions.

## INTRODUCTION

Metastatic spread is the leading cause of cancer-associated deaths. Metastases result from the shedding of circulating tumor cells (CTCs) from the primary tumor into the blood and subsequent establishment at distant organs [1]. Hence, CTCs may represent useful predictors of metastatic progression. Detection and analysis of CTCs and cell-free DNA (cfDNA) from the peripheral blood offers a minimally invasive procedure to test for tumor genotype [2]. cfDNA analytics allow the detection of chromosome arm-sized copy number alterations and point mutations [3, 4] and are of diagnostic use in the clinical context [5]. However, these approaches do not allow to enrich cell-free tumor DNA relative to cell-free normal DNA. Further, it remains unclear whether DNA fragments released from apoptotic or necrotic cells as cfDNA contain the same information as surviving tumor cells. The molecular profiling of CTCs therefore might provide information of tumor cells, including cells with metastatic potential that might not be identical with the information captured by cfDNA analytics. Besides, recent studies by Blogowski *et al*. [6, 7] found abnormal peripheral trafficking of bone marrow-derived stem cells in patients with gastric cancer but absent in other types of gastric neoplasms and healthy individuals, hence highlighting the potential of using these circulating bone marrow-derived stem cells as a biomarker. These liquid biopsies approaches may represent a valid alternative to tumor biopsies that are invasive, painful and provide only information for a small region of a tumor at a single time point. In addition, liquid biopsies could easily enable real-time monitoring of disease progression or treatment efficacy by repetitive sample collection of peripheral blood.

Cancer involves the accumulation of genomic alterations, starting from primary tumors to distant metastases. Due to the enormous progress made in personalized medicine, the choice of a targeted therapy for an individual patient is often made after analyzing the primary tumor for the expression and/or genomic status of a specific molecular target. However, metastatic tissue is often inaccessible and hence CTCs emerge as an alternative liquid biopsy that provides real-time molecular information of the metastatic tumor. This provides an opportunity for the clinician to select appropriate treatment regimens to target driver mutations at the right time that could help to improve the disease outcome. Although previous studies have shown that CTC counting was able to predict progression and overall survival of cancer patients, genomic analyses of CTCs could provide more information for personalized therapy [8]. At present, most of the CTC enumeration technologies that are established in research laboratories involve complicated equipment. This is a major drawback to the current CTC enumeration technologies as the operation of complex equipment has limited the usage of these technologies in hospitals. To address this, we have developed a simple, rapid and cost-efficient CTC capture system using a microfabricated silicon microsieve platform that requires minimal amount of blood (1-3 ml) [9].

Most studies on devices capturing CTCs have typically focused on platform development with limited insight on the molecular and functional characteristics of CTCs [10]. Major publications on the clinical utility of CTCs have focused on the relationship between CTC count and disease outcome [11–13]. However, it remains unclear how the quantification of CTCs can be used to guide treatment of cancer patients. This requires intervention studies with assignment of patients to different treatment groups based on CTCs count [14]. In addition, many of the existing CTC enrichment technologies rely on epithelial markers for isolation of CTCs, thereby missing CTCs that lost the epithelial signature when they have undergone epithelial-to-mesenchymal (EMT) transition [15]. Consequently, the genomics characterization of CTCs from both epithelial and mesenchymal traits might be required for a comprehensive assessment of genetic signatures of CTCs.

To address this, our microsieve filtration system separated CTCs from normal blood cells based on size differences [9, 16]. This size-based approach allowed us to isolate CTCs independently of their *EpCAM* expression, hence allowing the evaluation of the molecular profile of CTCs in a molecularly unbiased manner. We characterized the profiles of matched bulk primary tumors and enriched CTCs using an amplicon-based targeted sequencing approach on a custom-designed gene panel consisting of druggable or frequently mutated genes in colorectal cancer.

In this study, we describe our approach for the identification of somatic mutations present in the CTCs by minimizing the false-positive mutation detection in the amplified DNA. We present the genetic signatures and mutation spectrum of CTCs from colorectal cancer patients. Interestingly, our work offers novel insights by demonstrating that the frequencies of somatic mutations detectable in CTCs correlates with prognostic markers and their mutation signatures resemble the primary tumors’ signature. Importantly, our findings have strengthened the clinical utility of minimally invasive CTC analysis beyond the prediction of disease outcome based on CTC count in providing useful genetic signatures to guide the assignment of appropriate treatment for the cancer patients.

## RESULTS

We collected EDTA bloods and matched primary tumor and normal tissues from 48 colorectal cancer patients. The clinicopathological parameters of these patients are displayed in Table 1 and Supplementary Table 2. The CTCs were isolated using a size-based filtration system with a microfabricated silicon microsieve [9]. The DNA was extracted from CTCs, primary tumor and normal tissues. In order to get sufficient DNA for downstream analysis, whole genome amplification (WGA) was performed on the DNA extracted from CTCs (Supplementary Figure 1).

**Table 1:** Clinicopathological parameters of the samples recruited for this study

Clinical parameters	Number of patients
Age (Median), range	60, 26-84
Gender	
Male	27 (56.2%)
Female	21 (43.8%)
Site of primary tumor	
R (Ascending colon)	2
R (Caecum)	3
L (Descending colon)	4
L (Rectum)	22
L (Rectosigmoid)	4
L (Sigmoid)	11
L (Splenic flexure)	2
Dukes’ stage	
A	7
B	16
C	21
D	4
CEA level	
< 5ng/ml	15
≥ 5ng/ml	9
Not Available	24
Neo-adjuvant chemotherapy and radiotherapy	
Yes	9
No	39

### Evaluation of the amplification errors introduced by whole genome amplification (WGA)

DNA amplification with small amount of starting material is accompanied by a significant level of errors and biases that have made the data interpretation challenging [17]. In order to systematically evaluate the amplification errors introduced by WGA, we have performed WGA on normal tissue DNA collected from 14 patients using amounts of DNA that were comparable with the DNA derived from CTCs. We performed variant calling on the amplified normal tissue DNAs using the unamplified normal tissue DNA as reference. Hence, the variants detected in the amplified DNA samples represent errors introduced by the WGA. By evaluating the amplification errors collected from these 14 samples, we observed a combination of recurrent amplification errors as well as random non-recurrent errors with a median variant allele frequency of 0.5% and 1%, respectively (Supplementary Figure 2, Supplementary Table 3). In order to filter these amplification errors from the patient samples, we removed all the recurrent amplification errors from our dataset and set a threshold to only keep the variants with allele frequencies of >1%. In order to estimate the false positive rate for paired tumor matching mutations, we assessed the number of detected variants with allele frequency > 1% in the 14 amplified normal DNA samples that matched with the somatic mutations found in the paired tumor samples. We observed a false positive mutation match rate of 0.2% where a total of 4 out of 1,915 variants from the 14 amplified normal DNAs matched with variants found in the respective paired tumor samples, suggesting that 2 mutation calls out of 1,000 could match a tumor mutation by chance. Since we identified an average of 84 somatic mutations per CTC sample, the likelihood was low to misclassify CTC variants as tumor-derived mutations.

### The mutation profiles of primary tumor and CTCs

**Figure 1:**
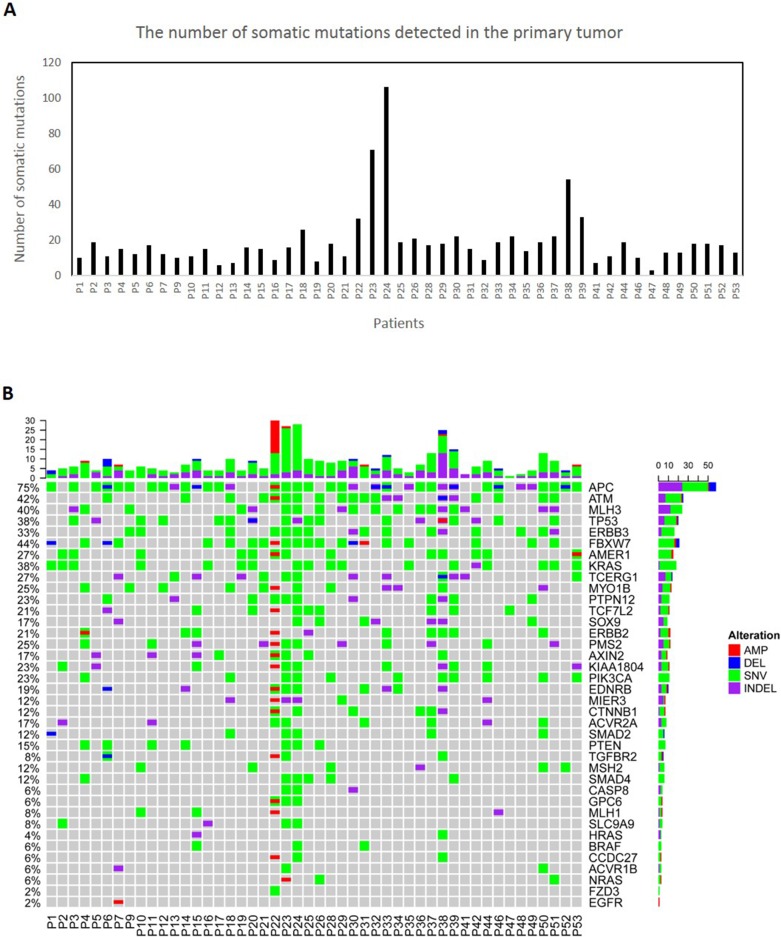
Molecular characterization of 48 primary colorectal tumors **(A)** The number of all the somatic mutations detected in the primary tumors. **(B)** The tabulation of exonic SNVs, in-frame or frame-shift Indels and CNVs of frequently mutated genes in the primary tumors. The upper panel displays the barplot of cumulative numbers of alterations for individual patients. The right panel displays the cumulative numbers of alterations for individual genes. The frequencies on the left display the percentages of samples where a gene is altered. Patient's IDs are shown at the bottom. Red rectangles represent amplifications. Blue rectangles represent deletions. Green rectangles represent missense, Stopgain or Stoploss somatic SNVs. Purple rectangles represent somatic Indels.

We have designed a gene panel consisting of 39 most frequently mutated and druggable genes in colorectal cancer [18] using Qiagen's GeneRead DNAseq Custom Builder tool. We performed targeted amplicon-sequencing on the matched CTCs, primary tumor and normal tissues with the median coverage of at least 400x (Supplementary Table 4). We found numerous somatic single nucleotide variants (SNVs), insertions/deletions (Indels) and copy number variations (CNVs) affecting the WNT (mutated *APC* & *TCF7L2*), RAS (mutated *KRAS, ERBB2* & *ERBB3*) and P53 (mutated *TP53* & *ATM*) signaling pathways in the primary tumors (Figure 1, Supplementary Tables 5 and 6).

**Figure 2:**
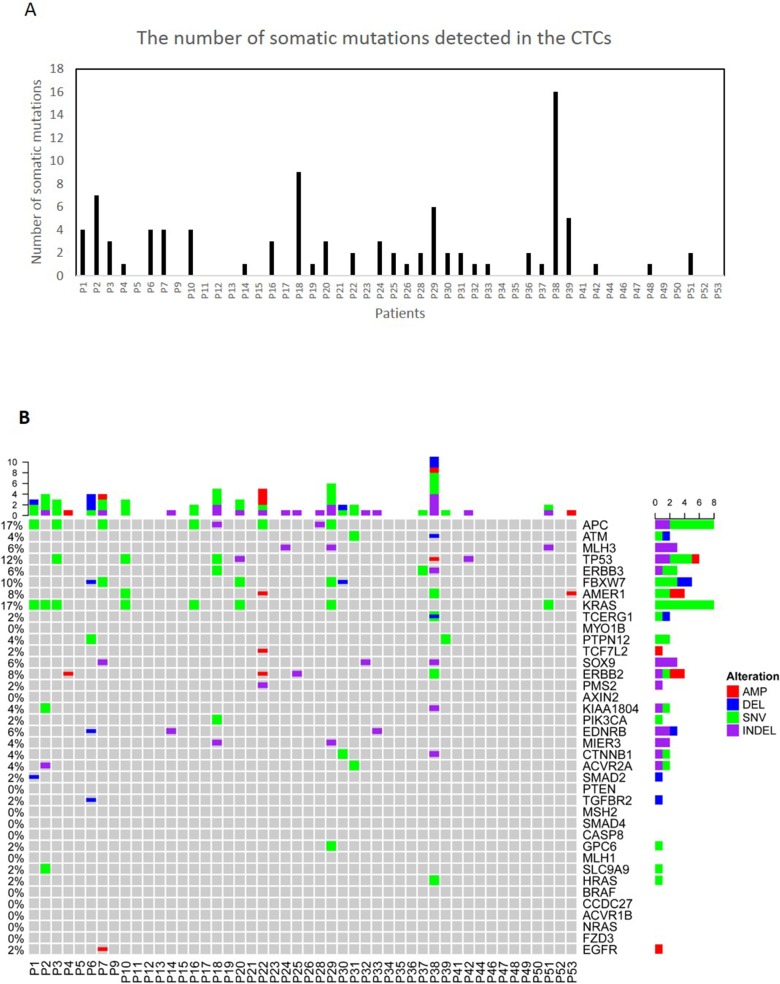
Molecular characterization of CTCs **(A)** The number of all the somatic mutations detected in the CTCs. **(B)** The tabulation of exonic SNVs, in-frame or frame-shift Indels and CNVs of frequently mutated genes of CTCs samples. Figure panels are described in Figure 1B.

Since false-positive variants could be detected in the amplified CTCs due to the amplification errors during WGA, we have only considered variants shared between the matched primary tumor and CTCs (Supplementary Tables 6 and 7). We found primary tumor matching somatic SNVs, Indels or CNVs in 60% of the CTC-enriched blood samples (Figure 2A). The lack of mutations in the remaining samples might be explained by the absence of CTCs in these samples, presence of CTCs with a frequency of <1% or tumor heterogeneity where CTCs were shed from a tumor subclone that is different from the part of the primary tumor that was sequenced. We did not observe common clinical features in the 40% of patients where no tumor-matching mutations were identified. We found frequent *APC, KRAS, ERBB3, TP53* and *FBXW7* mutations as well as the *ERBB2* amplification in our CTC samples (Figure 2B).

### The mutation profiling of CTCs allows the identification of genetic signatures of the disseminated tumor subclones

In order to eliminate false-positive variants in the amplified CTC DNA, we have only considered the shared mutation between the matched primary tumor and CTCs as genuine variants. With this conservative approach, we miss mutations present in the CTCs but undetected in the matched primary tumor. To investigate the quality of mutation calls that are specific for the CTC samples and not detected in the paired tumor, we performed two independent WGA experiments on the extracted CTC DNA material from 10 patients followed by panel sequencing. We reasoned that the shared variants detected in two independently processed samples that do not match recurrent errors from our earlier WGAs (see Methods) are unlikely false-positive variants introduced by the WGA. With this procedure, we found new variants in the CTC samples from five patients that were not detected in the matched primary tumors (Supplementary Table 8). We observed that the CTCs from patient P16 displayed a heterogeneous profile with many new mutations that were undetected in the matched primary tumor. In addition, we found a somatic mutation in *ERBB3* in the CTCs from patient P16 at a mean frequency of 8%. This indicated that besides the *KRAS G12D* driver mutation that was present in the respective primary tumor and CTCs, there was also a tumor subclone with an *ERBB3* mutation that was shed into the circulating blood. The mutations that were present in the CTCs but undetected in the primary tumor could be explained by tumor heterogeneity where the molecularly investigated part of the resected tumor only represents a small portion but not the whole tumor. Our result suggests that the mutation profiling of CTCs could provide information on the genetic signatures of disseminated tumor cells derived from a tumor subclone that could be missed by bulk tumor sequencing.

### The allele frequency of the somatic mutations in the CTCs correlates with CEA tumor marker levels

**Figure 3:**
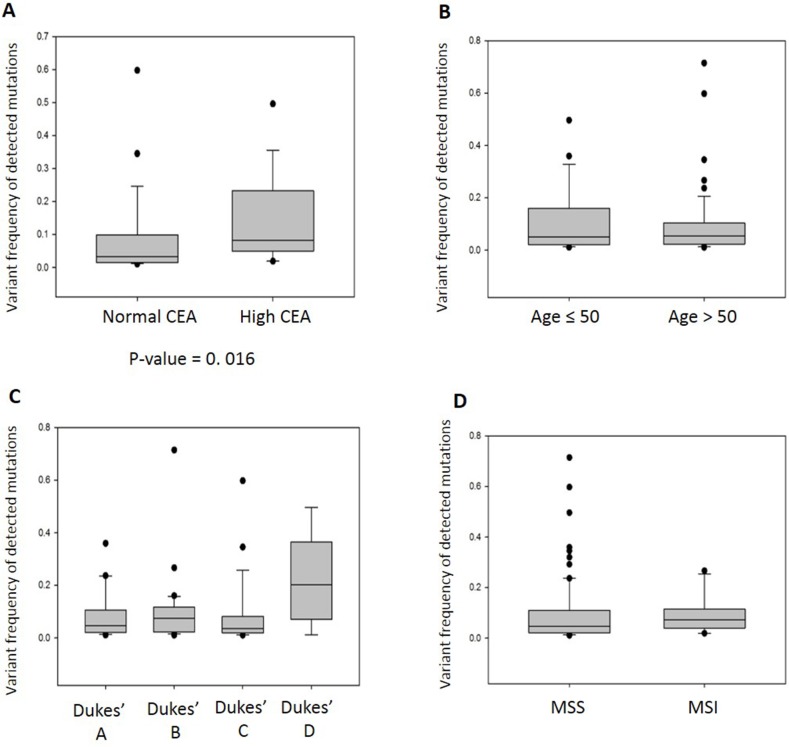
The allele frequencies of somatic mutations detected in CTCs of 48 colorectal cancer patients correlate with a prognostic marker **(A)** The somatic mutation frequency of CTCs is significantly associated with high CEA serum cancer biomarker (p-value=0.016, Mann-Whitney Rank Sum Test). **(B)** There is no association between the somatic mutation frequency and age of the patient. **(C)** Although there is insignificant association between the somatic mutation frequency and Dukes’ staging, we noted that there is higher mutation frequency in patients with Dukes’ D stage. **(D)** There is no significant difference between the somatic mutation frequency of patients with microsatellite instability (MSI) and microsatellite stable (MSS) tumors.

In order to evaluate the clinical utility of the somatic mutations found in the CTCs, we correlated the allele frequencies of the somatic mutations with the clinical parameters such as age, Dukes’ stage, CEA tumor marker level and microsatellite stability status. Though only half of the patient cohort has available CEA information, we observed significant correlation between the allele frequencies of somatic mutations and abnormal CEA level (> 5 ng/ml) (Figure 3A). The samples with high mutation frequency indicated the abundance of CTCs in circulation that is often associated with poor prognosis [19, 20]. Hence, our results suggested that allele frequencies of somatic mutations in CTCs have prognostic value. Although there was insignificant correlation between the frequency of somatic mutations with age and Dukes’ staging (Figure 3B–3C), we noted that there was higher mutation frequency in the patients with Dukes’ D stage. Dukes’ stage and CEA level are not independent from each other so that the correlation with mutation frequency are likely to have a common cause. Microsatellite instability (MSI) has been reported to be associated with high mutation frequency in colorectal cancer patients [18]. We did not observe significant differences between the mutation frequency detected in patients with MSI and with microsatellite stable (MSS) tumors (Figure 3D), probably due to the small sample size of patients with MSI.

### The somatic mutations detected in the CTCs display a mutation signature characteristic for colorectal cancer

**Figure 4:**
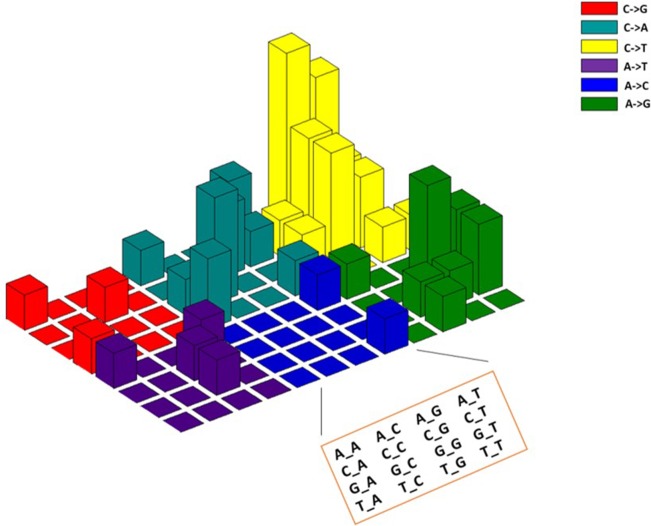
The mutation spectrum and signatures of CTCs of 48 colorectal cancer patients resembles the signatures reported in primary colorectal cancer tumors The mutation spectrum of somatic mutations found in CTCs displayed predominant C→T nucleotide changes that have been reported in colorectal cancer by Lawrence *et al* 2013 [23].

The accumulation of somatic mutations leading to the formation of cancer is either driven by the individual inherited genetic background, exposure to carcinogens or by chance [21]. Different cancer types display different mutation signatures that are associated with different factors such as aging, smoking or *BRCA1/2* mutations [22, 23]. We explored if the mutations detected in the CTCs displayed a signature that is related to colorectal cancer. Interestingly, we found the presence of a C→T mutation signature in our samples (Figure 4). This observation is consistent with a previous finding where C→T is the predominant mutation signature in colorectal cancer [23]. Further, the *APC* mutations that have been recognized as a hallmark of colorectal cancer [24] were detected frequently in the CTC samples. These findings demonstrate that the signatures of somatic mutations in CTC samples can resemble the primary tumor.

## DISCUSSION

Tumor biopsies and computerized topography (CT) scans are the standard of care in the clinic for obtaining tumor samples and monitor disease progression. However, these practices are invasive and can cause complications. The minimally-invasive liquid biopsy has emerged as the new alternative for the invasive and expensive procedures. In addition, liquid biopsies allow repetitive sample collection, hence permitting real-time monitoring of disease progression and treatment efficacy.

CellSearch^®^ remains the only FDA-approved test for capturing and enumerating CTCs to guide clinical decision. However, this platform relies on epithelial marker for the identification of CTCs, therefore missing the subset of CTCs that have lost the epithelial signature when undergoing EMT [25]. Importantly, the work by Yu *et al*. has demonstrated that CTCs from breast cancer patients exhibited dynamic changes in the epithelial and mesenchymal composition at different stages of disease progression [15]. In addition, the study by Markiewicz *et al*. has reported that CTCs with mesenchymal phenotype were significantly correlated with metastatic potential with lymph node involvement [26]. Hence, only evaluating CTCs of both epithelial and mesenchymal properties can provide a comprehensive overview of the genetic signatures of disseminated cells in cancer patients. To address this, we performed CTC isolation using a microsieve size filtration system that allows for enrichment of larger cells like CTCs, not restricting to epithelial CTCs. However, this approach is generally accompanied with high contamination of normal blood cells. To accommodate this, we employed deep targeted amplicon next generation sequencing (NGS) of frequently mutated genes in colorectal cancer. Though Heitzer *et al*. have previously reported mutation profiling of CTCs and tumors from colorectal cancer patients by deep amplicon sequencing, their study analyzed samples of only two patients [27]. Moreover, Heitzer *et al*. used the CellSearch^®^ platform for the isolation of EpCAM-positive CTCs, thereby omitting CTCs with mesenchymal properties. In contrast, our work involves a larger sample size of 48 patients, and we isolated CTCs based on size, not limited to CTCs with an epithelial signature.

In order to get sufficient DNA for downstream genomics analysis, whole genome amplification (WGA) is often used to amplify genomic DNA from limited material such as CTCs. However, the utilization of WGA has its limitation as it introduces errors, allelic drop-out and non-uniform amplification [28, 29]. In order to address this, we present a systematic analysis pipeline for the detection of somatic mutations in CTCs that minimizes false-positive mutation calls in the amplified DNAs.

In order to evaluate the clinical utility of CTCs as minimally invasive liquid biopsy in providing the mutation profiles of the matched tumor, we performed molecular characterization of CTCs using amplicon-based targeted sequencing approach on a panel of 39 druggable and frequently mutated genes in colorectal cancers [18]. We detected primary tumor matching mutations in 29 colorectal cancer patients (60%), demonstrating that the size-based CTC enumeration by the microsieve system can efficiently enrich CTCs from colorectal cancer patients. We provide evidence for the presence of key driver mutations in genes such as *APC, KRAS, ERBB3, TP53* and *FBXW7* in the disseminated CTCs. In addition, we observed *ERBB2* amplification in CTCs, which has been described in colorectal tumors [18]. Further, we suggest a workflow that allows the identification of new emerging mutations in CTCs that are undetected in the matched primary tumor, providing information on the tumor subclones that are responsible for distant metastases. This methodology is of particular interest for screening purposes and preoperative patient management. Characterizing the mutation profiles of CTCs can provide important information of the genetic signatures of disseminated tumor cells. This is critical as understanding the mutation profiles of disseminated tumor cells might provide the possibility to target distant metastases that represent the major cause of cancer-associated death. Importantly, some of the driver mutations present in the CTCs are targetable with available therapeutic agents such as *ERBB2* amplification that can be targeted with Trastuzumab. Molecular characterization of CTCs therefore provides an opportunity to suggest therapeutic agents to target CTCs and potential metastases.

We hypothesized that the mutation allele frequency of the disseminated CTCs could provide essential information on the tumor burden as disease surveillance marker. Strikingly, we found positive correlation between the frequencies of somatic variants detected in the CTCs with abnormal CEA level, providing evidence for the utility of CTCs as a biomarker in the clinic to monitor disease progression. Since our clinical samples were derived from newly resected patients that lack sufficient follow-up information, the correlation between the mutation frequency in CTCs and the disease outcome could not be determined and has to be done in future studies.

Interestingly, we found that the mutation spectrum of CTCs of colorectal cancer patients resembles the mutation signature of colorectal cancer, suggesting that the identification of mutation signatures of CTCs could help to identify the origin of the primary tumor site. Carcinoma of unknown primary site (CUP) constitutes approximately 3-5% of all newly diagnosed malignancies [30, 31], hence it is being recognized as one of common cancer diagnoses. The capability to identify the origin site of these neoplasms is an unmet need for effective therapy because a substantial fraction of the current treatment regimens require prior knowledge of the type and origin of the tumor. The identification of tissue-characteristic mutation signatures through targeted sequencing of CTCs, as shown in the present study, provides an opportunity to clarify some CUP cases. More studies with larger sample sizes and broader targeted genomic regions across different cancer types are needed to validate this finding.

The work by Misale *et al*. [2] and Bettegowda *et al*. [4] have reported that the molecular profiling of cfDNA could provide genetic information to guide the selection of appropriate therapy. Though we could not perform the panel sequencing on the matched cfDNA since plasma DNA was not collected as part of this study, the work by Rothwell *et al*. [32] has demonstrated that the molecular profile of CTCs and cfDNA are comparable, hence the analysis of CTCs and cfDNA can provide similar information. It remains to be determined, for which cancer types and disease stages or monitoring situations analyses of cfDNA or CTCs are of advantage.

Cumulatively, we present an analysis pipeline for detection of somatic mutations in amplified CTC DNA by minimizing false-positive calls introduced by WGA. Our work represents the first report describing the molecular profile of CTCs from colorectal cancer in a comprehensive manner without limiting to CTCs with an epithelial phenotype. We show that the molecular analysis of CTCs has potential as a prognostic marker and provides useful information that can be linked to targeted therapy. Further, we propose that sequencing of pooled CTCs allows us to define mutation signatures that can resemble the signature of the tumor of origin. Our findings provide evidence for the clinical utility of CTCs that is essential to bring the concept of liquid biopsy to clinical implementation.

## MATERIALS AND METHODS

### Patients

A total of 48 patients diagnosed with colorectal cancer at the Concord Cancer Hospital Singapore were recruited. All patients have given written consent to participate in this study and the biological samples were collected from the patients following the protocols approved by the Institutional Review Board (IRB). The paired tumor and normal frozen tissues were obtained from the surgical resections and stored at −80°C. Blood samples for CTC enrichment were drawn before surgery and CEA measurements were taken at disease diagnosis.

### Isolation of CTCs

The pre-operative EDTA blood was collected from the patients and subjected to CTC isolation within six hours. CTCs were isolated using a size-based filtration system on a silicon microsieve platform as described previously [9]. Briefly, a total of 3 ml of blood was loaded on the IBN microsieve followed by 3 to 4 washes (1x PBS, 0.5% BSA, 2 mM EDTA) at a flow rate of 0.5 ml/min. The microsieve containing the isolated CTCs was retrieved from the cartridge and stored at −80°C until further use.

### DNA extraction

The DNA was extracted from the frozen tissues using DNeasy blood and tissue kit (Qiagen), while the DNA from CTCs was extracted using the QIAamp DNA mini kit (Qiagen) following the manufacturer's protocol. Briefly, 200 μl of PBS with proteinase K was applied to the tube containing 15-25 mg of tissues or isolated CTCs, followed by addition of lysis buffer and incubation at 56°C for one hour. The solution containing the DNA from the lysed tissues and CTCs was placed into the spin column and washed with Buffers AW1 and AW2 (Qiagen). The DNA was eluted in Buffer AE (Qiagen) and its concentration was assayed using NanoDrop 1000 spectrophotometer (Thermo Scientific).

### Whole genome amplification

Whole genome amplification was performed on the extracted DNA from the CTC samples using REPLI-g UltraFast mini kit (Qiagen). The denaturation buffer was added to the DNA followed by a 3 min incubation at room temperature. The denaturation was terminated by addition of neutralization buffer. The DNA amplification was performed in a reaction mix consisting of reaction buffer and DNA polymerase for 1.5 hours at 30°C. The reaction was terminated by inactivation of the DNA polymerase at 65°C for 3 min. The amplified DNA was cleaned up using ethanol precipitation. DNA concentration was assayed using Qubit fluorometer (Thermo Scientific).

### GeneRead targeted DNAseq

We have custom designed a gene panel using Qiagen's GeneRead DNAseq Custom Builder tool. This GeneRead panel generated PCR products of ∼150bp and consisted of 39 most frequently mutated or druggable genes in colorectal cancer as reported by TCGA [18] with a targeted region of ∼110kb. The list of genes and its coverage regions are listed in Supplementary Table 1. Multiplex PCR was performed using GeneRead HotStar Taq DNA polymerase and four primer pools with a total of 80 ng input DNA. The amplicons were pooled together and cleaned using AMPure beads (Beckman Coulter). The PCR-enriched DNA was subjected to next-generation sequencing library construction using GeneRead DNA library core kit (Qiagen). Each library was barcoded with a unique index and quantified using KAPA Library Quantification kit (Kapa Biosystems). Equal amounts of individual libraries were pooled together for a 150bp paired-end sequencing run on the HiSeq 2500 (Illumina) platform.

### Reads alignment and base quality refinement

The sequenced reads were mapped to the human reference genome hg19 using BWA pipeline 1.1 [33]. The aligned reads were sorted based on coordinates. Since the length of the PCR products were generally small with a median size of 158bp, the output of paired-end 150bp sequencing run generated overlapping reads for majority of the PCR products. In order to take advantage of these overlapping reads to improve the sequencing accuracy, we increased the base quality of those consistent overlapping bases and decreased the base quality of the inconsistent overlapping bases. We trimmed the 5’ end of reads if they matched (allowed up to maximum 20% mismatch) to any of the PCR primers designed for the same chromosome. We realigned the reads around Indels and recalibrated base quality scores using Genome Analysis Toolkit 3.3 [34, 35].

### Variant calling and mutation spectrum analysis

We used LoFreq 2.1.1 pipeline for detection of single nucleotide variants (SNVs) and insertions/deletions (Indels) variants with default parameters [36]. We used Quandico 1.13 [37] for copy number variants (CNVs) detection with the following modification to the default setting: primer length was set to 21 (average primer length of our GeneRead panel); reads with mapping quality score less than 30 were excluded from the analysis and we grouped the reads into regions as qcluster. We plot the mutation spectrum chart by computing the frequency of various nucleotide changes in the detected SNVs.

### Microsatellite instability (MSI) analysis

We performed MSI analysis on the tumor DNA materials using MSI Analysis kit (Promega) following the manufacturer's recommendations. Briefly, DNA from matched normal and tumor specimens were amplified using a panel of markers designed to amplify targeted microsatellite regions (BAT-25, BAT-26, NR-21, NR-24 and MONO-27). A reaction mix consists of Gold STAR 10x buffer, MSI 10x Primer Pair Mix and AmpliTaq Gold DNA polymerase was added into 2ng of DNA followed by amplification on the thermal cycler under the following conditions: 95°C for 11 min, 96°C for 11 min; 10 cycles of 94°C for 30 sec, ramp to 58°C in 68 seconds and hold for 30 sec, ramp to 70°C in 50 sec and hold for 1 min, 20 cycles of 90°C for 30 sec, ramp to 58°C in 60 sec and hold for 30 sec, ramp to 70°C in 50 sec and hold for 1 min; 60°C for 30 min. The amplified fragments were analyzed on ABI PRISM^®^ 310 Genetic Analyzer (Thermo Fisher).

## SUPPLEMENTARY FIGURES AND TABLES




