# Supplementary material for: Molecular characterization of circulating colorectal tumor cells defines genetic signatures for individualized cancer care
Source: Oncotarget. 2017 Jul 10;8(40):68026–37. doi: 10.18632/oncotarget.19138 (PMC5620233; doi:10.18632/oncotarget.19138)
Supplement: Supplementary file 1 [file oncotarget-08-68026-s001.pdf]

Molecular characterization of circulating colorectal tumor cells defines genetic signatures for individualized cancer care

SUPPLEMENTARY MATERIALS

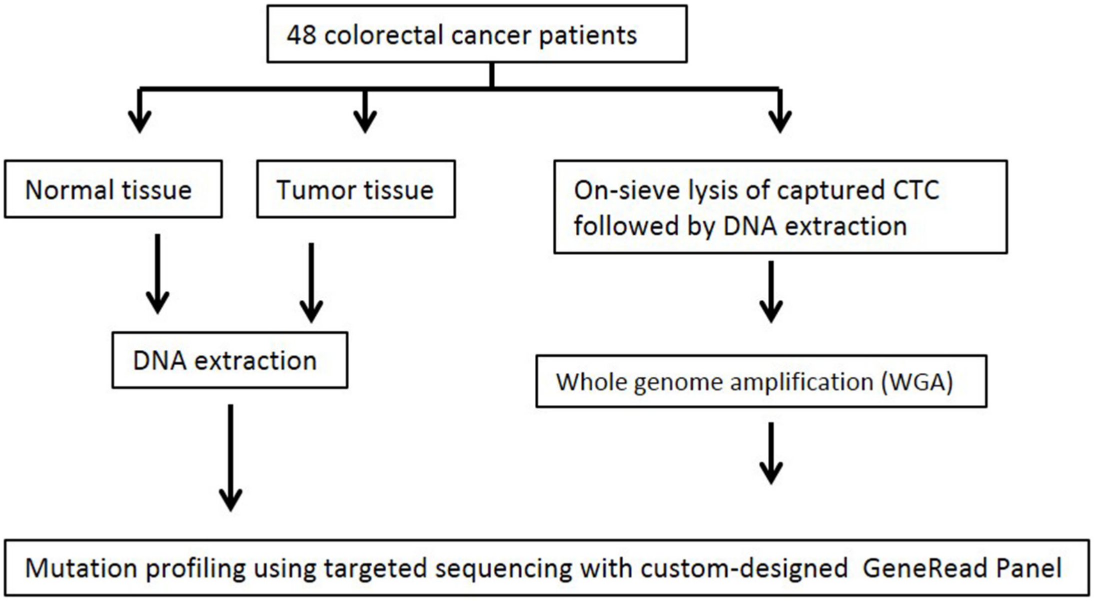

Supplementary Figure 1: The flowchart for the experimental set-up applied in this study.

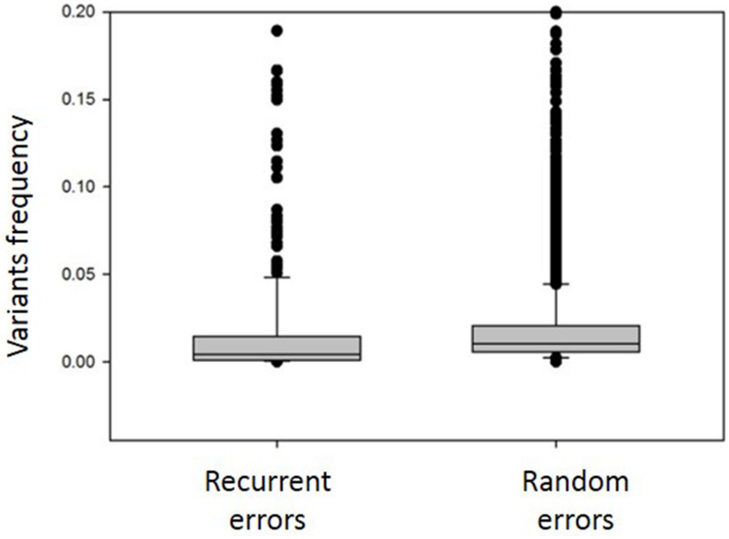

Supplementary Figure 2: The tabulation for the frequencies of variants detected in the amplified samples due to amplification errors.

Supplementary Table 1: The list of 39 genes and its coverage regions for the GeneRead Targeted DNAseq panel.

See Supplementary File 1

Supplementary Table 2: The clinicopathological parameters of the samples recruited for this study.

See Supplementary File 1

Supplementary Table 3: The WGA errors found in 14 amplified normal tissue samples.

See Supplementary File 1

Supplementary Table 4: The tabulation for the sequencing coverage of all samples.

See Supplementary File 1

Supplementary Table 5: The SNV and Indel mutations found in the primary tumors.

See Supplementary File 1

Supplementary Table 6: The CNVs found in the primary tumors and CTCs samples.

See Supplementary File 1

Supplementary Table 7: The SNV and Indel mutations found in the CTCs samples.

See Supplementary File 1

Supplementary Table 8: Somatic variants found in CTC samples with two independent WGA experiments.

See Supplementary File 1
